# Induction of Ovarian Leiomyosarcomas in Mice by Conditional Inactivation of *Brca1* and *p53*

**DOI:** 10.1371/journal.pone.0008404

**Published:** 2009-12-31

**Authors:** Bridget A. Quinn, Tiffany Brake, Xiang Hua, Kimberly Baxter-Jones, Samuel Litwin, Lora Hedrick Ellenson, Denise C. Connolly

**Affiliations:** 1 Fox Chase Cancer Center, Philadelphia, Pennsylvania, United States of America; 2 NewYork-Presbyterian Hospital, Weil Medical College of Cornell University, New York, New York, United States of America; Cincinnati Children's Research Foundation, United States of America

## Abstract

**Background:**

Approximately one out of every ten cases of epithelial ovarian cancer (EOC) is inherited. The majority of inherited cases of EOC result from mutations in the breast cancer associated gene 1 (*BRCA1*). In addition to mutation of *BRCA1*, mutation of the *p53* gene is often found in patients with inherited breast and ovarian cancer syndrome.

**Methodology/Principal Findings:**

We investigated the role of loss of function of *BRCA1* and *p53* in ovarian cancer development using mouse models with conditionally expressed alleles of *Brca1* and/or *p53*. Our results show that ovary-specific Cre-recombinase-mediated conditional inactivation of both *Brca1^LoxP/LoxP^* and *p53^LoxP/LoxP^* resulted in ovarian or reproductive tract tumor formation in 54% of mice, whereas conditional inactivation of either allele alone infrequently resulted in tumors (≤5% of mice). In mice with conditionally inactivated *Brca1^LoxP/LoxP^* and *p53^LoxP/LoxP^*, ovarian tumors arose after long latency with the majority exhibiting histological features consistent with high grade leiomyosarcomas lacking expression of epithelial, follicular or lymphocyte markers. In addition, tumors with conditional inactivation of both *Brca1^LoxP/LoxP^* and *p53^LoxP/LoxP^* exhibited greater genomic instability compared to an ovarian tumor with inactivation of only *p53^LoxP/LoxP^*.

**Conclusions/Significance:**

Although conditional inactivation of both *Brca1* and *p53* results in ovarian tumorigenesis, our results suggest that additional genetic alterations or alternative methods for targeting epithelial cells of the ovary or fallopian tube for conditional inactivation of *Brca1* and *p53* are required for the development of a mouse model of *Brca1-*associated inherited EOC.

## Introduction

Approximately 10% of cases of epithelial ovarian cancer (EOC) are associated with a clear hereditary predisposition to disease; the vast majority of cases resulting from inherited alterations in the breast cancer-associated tumor suppressor genes 1 or 2 (*BRCA1* or *BRCA2*) (reviewed in: [1], [2], [3]). *BRCA1* and *BRCA2* were originally identified based on genetic linkage to families with an increased risk of breast and ovarian cancer. The proteins encoded by these genes control normal cellular growth by their involvement in DNA damage repair, maintenance of genomic integrity, chromatin remodeling and transcription regulation [4], [5]. Linkage to the *BRCA1* gene exists in ∼80% of families with inherited risk of breast and ovarian cancer [1], [2], [3]. Although mutations of the *BRCA1* gene are not frequently detected in sporadic cases of EOC, recent studies have suggested that other mechanisms of inactivation of this tumor suppressor gene, such as promoter methylation and mutations in non-coding regions that effect functional protein expression, may exist in sporadic tumors [6], [7], [8], [9], [10]. In addition to alterations in *BRCA1* and *BRCA2*, mutation of the *p53* gene is reported in 70–90% of patients with familial breast and ovarian cancer syndrome [11], [12], [13], [14]. Interestingly, *p53* mutations are identified more frequently in *BRCA1*-associated and sporadic serous ovarian carcinomas than in sporadic ovarian cancers of other histologic subtypes [11], [12], [13], [14]. A role for *p53* in *BRCA1*-associated inherited breast and ovarian cancer is further supported by the cooperation of *p53* with *BRCA1* in the development of mammary tumor development in genetically engineered mouse (GEM) models [15], [16].

Despite recent advances in the development of GEM models of EOC [17], [18], [19], [20], [21] and the existence of mouse models of *Brca1*-associated inherited breast cancer [15], [16], analogous mouse models that develop *Brca1*-associated inherited invasive EOC have been more difficult to develop. The delay in developing such a model owes largely to controversy over the identity of the cell type of origin of *BRCA1*-associated inherited invasive EOC and the resultant difficulty in targeting molecular alterations to the correct cell type. Because homozygous deletion of *Brca1* leads to early embryonic lethality in mice, attempts to establish such a model have employed strains of mice with conditional inactivation of *Brca1*. Two previous studies [22], [23] investigated the effects of ovary-specific conditional inactivation of *Brca1* using distinct genetically engineered strains of mice harboring LoxP-flanked alleles of *Brca1* [16], [24]. In these studies, ovary-specific Cre-recombinase expression was achieved by genetic means [22] or by direct administration of adenovirus encoded *Cre-recombinase* [23]. In both cases, ovary-specific conditional inactivation of *Brca1* led to the development of pre-neoplastic [23] or benign [22] epithelial lesions in the ovary, but in neither case were invasive ovarian cancers observed. A separate study, using an *ex vivo* retroviral transduction strategy for conditional inactivation of LoxP-flanked *Brca1* and *p53* alleles in ovarian explants, showed that inactivation of both *Brca1* and *p53* in conjunction with expression of *Myc* led to transformation of ovarian cells and that these cells were tumorigenic in recipient mice [25]. In the present study, we sought to extend these previous observations by testing whether conditional inactivation of *Brca1* and *p53* in the ovarian surface epithelium *in situ* is sufficient to establish an autochthonous mouse model of *BRCA1*-associated invasive EOC. To this end, we used previously established strains of genetically engineered mice harboring LoxP-flanked *Brca1* [16] in conjunction with mice harboring LoxP-flanked *p53* [26] or a missense gain-of-function mutant allele of *p53* [27] that is analogous to the R175H hotspot mutation frequently identified in human cancers [28], [29], [30].

## Results

### Intrabursal Administration of Adenovirus Results in Efficient Infection of Ovarian Surface Epithelial Cells

**Figure 1:**
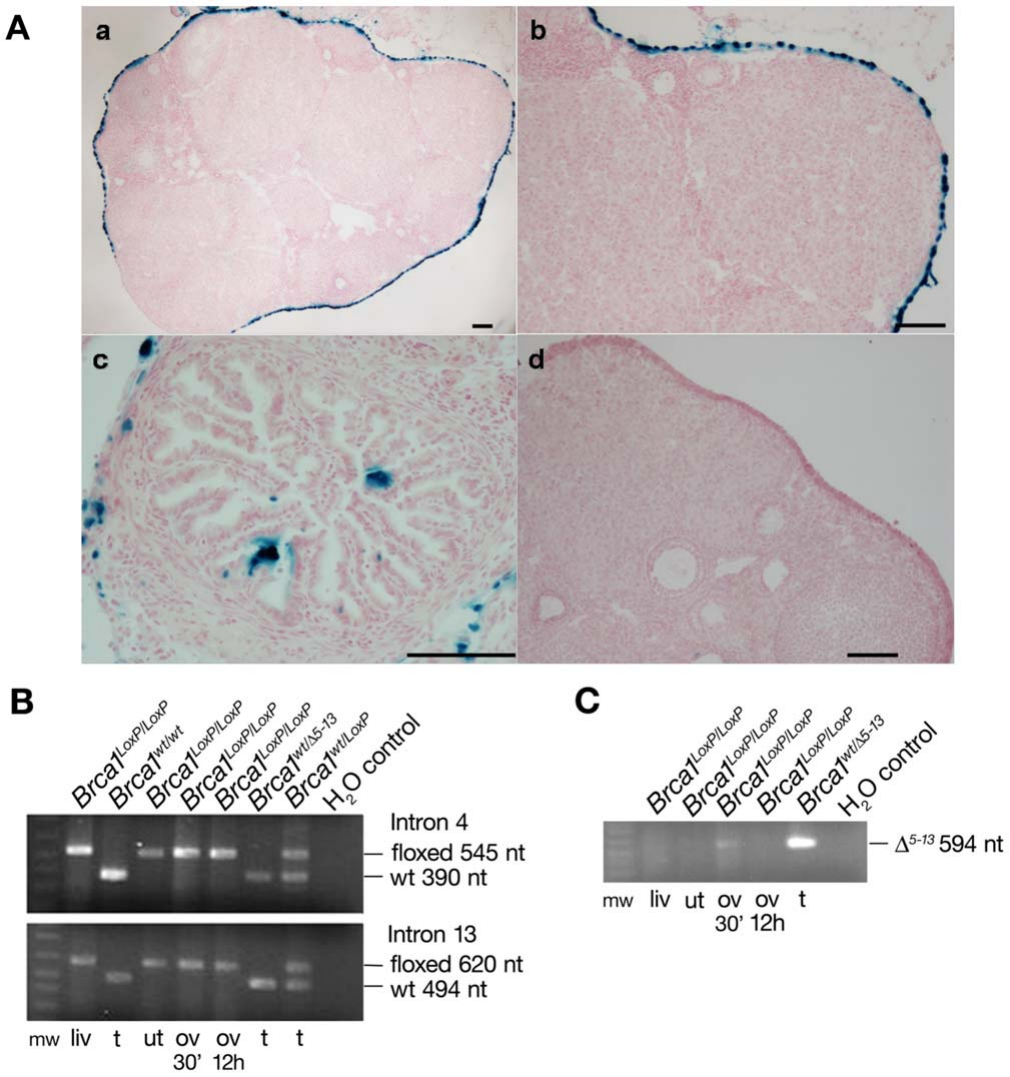
Intrabursal injection of Adenovirus results in ovarian surface epithelial cell-restricted infection. A) Histochemical detection of β-galactosidase expression in tissue sections of ovaries of a wild type mouse that received intrabursal injection of Ad5-CMV-LacZ show staining of the ovarian surface epithelium and absence of staining of the underlying ovarian cortex (panels a and b). Occasional β-galactosidase positive cells were also observed in fallopian tube (panel c) while no staining was observed in a control mouse injected with PBS (panel d). Scale bars in each image represent 100 µm. B) PCR amplification of LoxP containing sites in introns 4 (top) and 13 (bottom) of *Brca1* in *Brca1^LoxP/LoxP^*, *Brca1^wt/wt^*, *Brca1^wt^* ^/Δ5-13^ and *Brca1^WT/LoxP^* mice. Genomic DNA was isolated after digestion with proteinase K from the following tissues: liv–liver, t–tail, ut–uterus, ov 30′–ovary after 30 minute digestion and ov 12 h–ovary after 12 hours of digestion. The sizes of the LoxP flanked and wild type (wt) intron 4 and 13 PCR products are indicated. C) PCR amplification of 594 nt excised *Brca1* ^Δ5-13^ allele was detected only in the ovary exposed to digestion for 30 minutes and in the genomic DNA isolated from a *Brca1^wt^* ^/Δ5-13^ mouse.

Because *Brca1* ^−/−^ (knockout) mice are embryonic lethal and *p53* ^−/−^ mice develop and succumb from tumors (e.g., lymphoma, sarcoma, etc.) at young ages, the present study utilized genetically engineered mouse strains that express *LoxP* recombination sites within the *Brca1* and *p53* alleles, thus allowing conditional inactivation of each allele upon exposure to Cre-recombinase [31]. Since there are no existing mouse models that exhibit ovarian surface epithelium (OSE)-restricted expression of Cre-recombinase, we chose a viral approach for conditional Cre-mediated inactivation of LoxP-flanked alleles by intrabursal administration of Adenovirus-Cre. To demonstrate our capability to successfully perform intrabursal injections that result in OSE-restricted viral infection, we performed pilot experiments in which 8 week-old female C57Bl/6 mice received intrabursal injections of Ad5-CMV-ntLacZ as previously described [18], [19], [21], [23]. To test the extent and localization of viral infection, eight week-old female C57Bl/6 mice were given intrabursal injections of 5×10^7^ p.f.u. of Ad5-CMV-ntLacZ and euthanized one week post-surgery. Ovaries were dissected, snap frozen, sectioned and stained for β-galactosidase expression. Single intrabursal injection of Ad5-CMV-ntLacZ resulted in high efficiency, tissue restricted infection of the OSE with little or no infection of the underlying ovarian stromal tissue as shown by the β-galactosidase staining of thin sections of ovary (Figure 1A, panels a and b). Although staining was predominantly confined to the OSE, occasional β-galactosidase positive cells were observed in the fallopian tube (1A, panel c). No evidence of β-galactosidase staining was detected in the uterus or other organs indicating that viral infection had not spread beyond the site of injection (data not shown).

### Cre-LoxP-Mediated Conditional Inactivation of *Brca1*

To confirm infection with Ad5-CMV-Cre and tissue-restricted Cre-mediated excision of LoxP-flanked sequences, *Brca1^LoxP/LoxP^* mice received intrabursal injection of Ad5-CMV-Cre and were euthanized one week post-surgery and the ovaries, uterus and liver were excised. Because the OSE is a comparatively small component of the total ovary, detection of Cre-mediated excision of LoxP flanked sequences in the ovaries of *Brca1^LoxP/LoxP^* mice would likely be obscured if genomic DNA from the entire ovary was used for PCR amplification and detection of excision. To circumvent this issue, we prepared a tissue lysate enriched for digested OSE by limiting the initial digestion time of ovary specimens to 30 min. The remainder of the undigested ovary was then digested overnight. Genomic DNA was isolated from each tissue and PCR amplified using both the forward and reverse primers for *Brca1* introns 4 and 13. Cre-mediated excision of LoxP-flanked sequences of *Brca1^LoxP/LoxP^* was detected by amplification with Brca1-int4-Fwd and Brca1-int13-Rev primers. As expected, PCR amplification resulted in detection of LoxP flanked sequences in all tissues of the *Brca1^LoxP/LoxP^* mice (Figure 1B). Importantly, the excised *Brca1^Δ5-13^* product was detected only after short (30 min) digestion of the ovary and not in the liver, uterus or the remainder of the ovary specimens that were digested overnight (Figure 1C). These results confirm successful infection of the ovary by Ad5-CMV-Cre and Cre-mediated excision of LoxP-flanked *Brca1* sequences. Moreover, the absence of the excised *Brca1^Δ5-13^* product in the ovary specimens digested overnight (Figure 1C) suggests that the Ad5-CMV-Cre infection is limited to the outer part of the ovary (the OSE) without substantial penetration of the ovarian cortex or infection of uterus or other peritoneal organs.

Based on the successful demonstration of efficient, tissue-restricted infection with the Ad5-CMV-ntLacZ and Ad5-CMV-Cre viruses, we commenced with intrabursal Ad5-CMV-Cre injection of the following groups of mice: 1) *Brca1^LoxP/LoxP^*, 2) *p53^LoxP/LoxP^*, 3) *Brca1^LoxP/LoxP^;p53^LoxP/LoxP^*, 4) *Brca1^LoxP/LoxP^;p53^+/515A^* and 5) *Brca1^LoxP/LoxP^;p53^LoxP/515A^*. The total number of mice receiving bilateral or unilateral injection of Ad5-CMV-Cre or unilateral injection of PBS (control) for each group is summarized in Table 1.

**Table 1:** Summary of tumor incidence following intrabursal injection of Ad5-CMV-Cre recombinase.

Genotype	Total number of mice evaluated	Mice with any type of tumor (%)	Tumors with Cre-mediated conditional inactivation (%)*
***Brca1^LoxP/LoxP^***			
Bilateral Ad-Cre	40	9 (23)	0
Unilateral Ad-Cre	5	0	0
PBS	6	1 (17)	0
***p53^LoxP/LoxP^***			
Bilateral Ad-Cre	41	2 (5)	2 (5)
Unilateral Ad-Cre	6	1 (17)	0
PBS	6	0	0
***Brca1^LoxP/LoxP^;p53^LoxP/LoxP^***			
Bilateral Ad-Cre	35	20 (57)	19 (54)
Unilateral Ad-Cre	6	4 (66)	4 (66)
PBS	6	0	0
***Brca1^LoxP/LoxP^;p53^515C/WT^***			
Bilateral Ad-Cre	44	7 (16)	0
Unilateral Ad-Cre	6	2 (33)	0
PBS	6	0	0
***Brca1^LoxP/LoxP^;p53^515C/LoxP^***			
Bilateral Ad-Cre	15#	3 (20)	2 (13)
Unilateral Ad-Cre	3#	0	0
PBS	2#	0	0

* Confirmed by PCR amplification of excised product.

# Accrual terminated based on results from prior groups.

### Conditional Loss of *Brca1* or *p53* Alone Infrequently Results in Ovarian Tumor Formation

**Figure 2:**
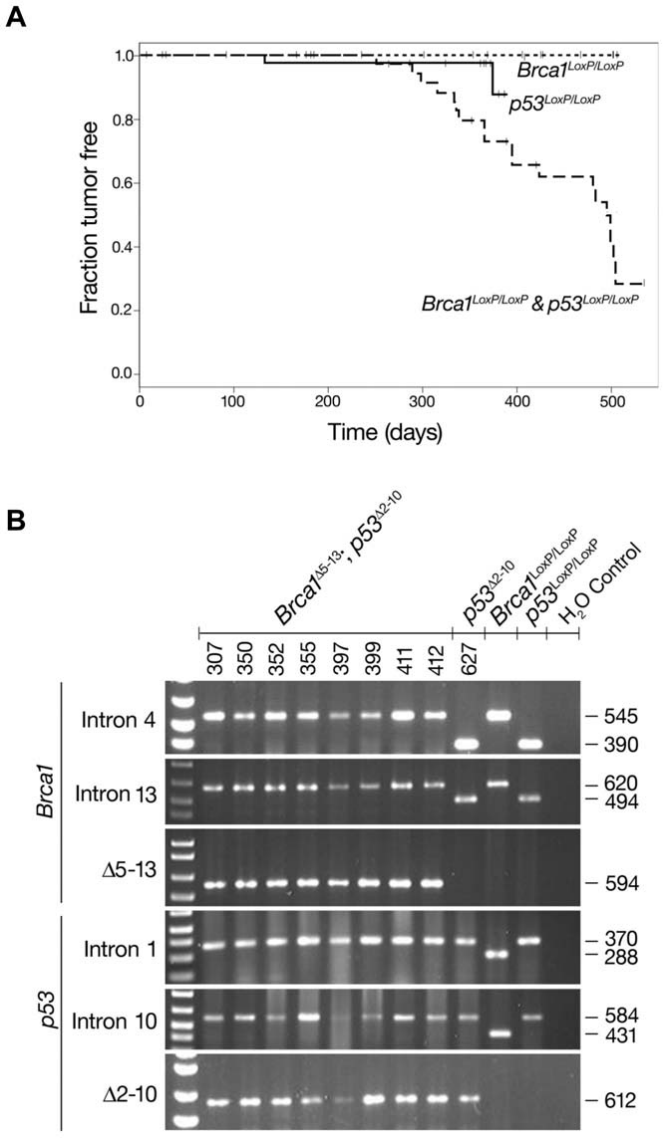
Incidence and latency of reproductive tract tumors in female mice with conditional inactivation of LoxP flanked alleles of *Brca1* and/or *p53*. A) Fraction of tumor free mice after intrabursal injection of Adenovirus-Cre recombinase. B) Genotyping analysis of DNA isolated from *Brca1* ^Δ5-13^;*p53* ^Δ2-10^ and *p53^Δ2-10^* ovarian tumors. Both the Lox P flanked and excised *Brca1* and *p53* alleles were amplified from *Brca1* ^Δ5-13^;*p53* ^Δ2-10^ tumors (cases 307, 350, 352 355, 397, 399, 411 and 412) and the Lox P flanked and excised *p53* allele was amplified from the single *p53^Δ2-10^* ovarian tumor (case 627). In each panel the left lane contains a DNA ladder and the sizes of the amplified products (nt) are indicated at the right.

A total of 51 *Brca1^LoxP/LoxP^* mice were evaluated (see Table 1 and Figure 2A), including 40 mice that received bilateral and 5 mice that received unilateral injection of Ad5-CMV-Cre as well as 6 unilateral PBS injected controls. The reproductive tracts and pathologically altered organs were isolated from all of these mice and subjected to histopathological evaluation (by L.H.E.). Of these 51 mice, 9/40 mice (23%) that received bilateral Ad5-CMV-Cre injection and 1/6 (17%) mice that received unilateral PBS injection developed tumors (Table 1) after long latency (i.e., average = 491±26 days after Ad5-CMV-Cre injection). No ovarian or reproductive tract tumors were detected (Figure 2A), and 8/9 tumors arising in bilaterally injected mice and the single tumor arising in a PBS injected mouse were extra-peritoneal. Because the tumors were located at sites distant from the site of Ad5-CMV-Cre injection, we considered the possibility that intrabursal injection of virus was leaky resulting in local or systemic infection beyond the original site of injection. To test this experimentally, genomic DNA was isolated from each of these tumors and subjected to PCR amplification of LoxP-flanked *Brca1* sequences in introns 4 and 13 and the excised *Brca1^Δ5-13^* product. Importantly, the *Brca1^Δ5-13^* excision product was not detected in any of the tumors identified in the *Brca1^LoxP/LoxP^* mice (Table 1 and data not shown). These results suggest that the tumors observed were sporadic and unrelated to the Cre-mediated excision of *Brca1^LoxP/LoxP^* resulting from local or systemic viral infection. The lack of ovarian tumors is consistent with previous studies [22], [23], [25] demonstrating that inactivation of *Brca1* alone is insufficient for malignant transformation in the mouse ovary.

Among the *p53^LoxP/LoxP^* mice evaluated in this study (n = 53), 41 received bilateral and 6 received unilateral injections of Ad5-CMV-Cre and 6 received unilateral injection with PBS (Table 1). None of the control PBS injected mice developed tumors, but a low frequency of tumor formation (3/53 mice) was observed in Ad5-CMV-Cre injected mice (Table 1 and Figure 2A). Specifically, one mouse developed a large palpable ovarian mass 132 days after bilateral Ad5-CMV-Cre injection, one mouse developed a small mass at the end of one of the uterine horns 373 days after unilateral injection of Ad5-CMV-Cre, and one control mouse developed a palpable subcutaneous mass located near the scapulae 353 days after PBS injection. Analysis of the genomic DNA isolated from the ovarian and uterine tumors resulted in amplification of the excised *p53^Δ2-10^* product (Figure 2B and data not shown), whereas no excision product was amplified from the DNA isolated from the subcutaneous scapular tumor. These results suggest that conditional inactivation of *p53* alone rarely results in ovarian tumor development (1/47 cases, 2%). This finding is again consistent with a previous study [19] reporting low frequency of ovarian tumor development (2/31 cases, 6%) after intrabursal Adenovirus-cre mediated conditional inactivation of *p53*.

### Conditional Loss of Both *Brca1* and *p53* Cooperates to Induce Ovarian Tumors

To determine whether inactivation of both *Brca1* and *p53* in the OSE can cooperate to induce ovarian tumorigenesis, we evaluated ovarian tumor formation in *Brca1^LoxP/LoxP^;p53^LoxP/LoxP^*, *Brca1^LoxP/LoxP^;p53^+/515A^* and *Brca1^LoxP/LoxP^;p53^LoxP/515A^* mice following intrabursal injection of Ad5-CMV-Cre. It is important to note that the *p53^515A^* mice used in the current study constitutively expressed the arginine to histidine substitution at amino acid 172 of *p53*. As previously reported [27], mice harboring the homozygous constitutively expressed mutant *p53^515A/515A^* allele developed tumors with an incidence, latency and spectrum (e.g., lymphomas and sarcomas) similar to that of homozygous *p53* knockout mice [32], [33]. Ovarian tumors were not detected in any of the *Brca1^LoxP/LoxP^;p53^+/515A^* and *Brca1^LoxP/LoxP^;p53^LoxP/515A^* mice at the time of euthanasia; therefore, these two groups were not informative for this study.

A total of 47 *Brca1^LoxP/LoxP^;p53^LoxP/LoxP^* mice were evaluated. Among these, 35 received bilateral Ad5-CMV-Cre injection, 6 received unilateral Ad5-CMV-Cre injection and 6 control mice were injected with PBS (Table 1). Tumors were detected in 20/35 (57%) mice with bilateral and 4/6 (66%) mice with unilateral injection of Ad5-CMV-Cre (Table 1 and Figure 2A) after long latency (409±81 days after Ad5-CMV-Cre injection). Pair-wise log-rank tests showed that *Brca1^LoxP/LoxP^;p53^LoxP/LoxP^* mice had significantly shorter average survival after conditional inactivation of LoxP flanked alleles than *Brca1^LoxP/LoxP^* or *p53^LoxP/LoxP^* mice (p<0.000001 and p<0.05 respectively). Among the 24 total tumors, we were able to demonstrate Cre-mediated excision of LoxP flanked sequences of both *Brca1^LoxP/LoxP^* and *p53^LoxP/LoxP^* in 23 cases (Table 2, Figure 2B and data not shown). The majority (22/23) of the tumors detected in *Brca1^LoxP/LoxP^;p53^LoxP/LoxP^* mice were associated with the ovaries and reproductive tract, and all exhibited Cre-mediated excision of LoxP flanked sequences (Table 2). Among these, there were 7 leiomyosarcomas of the ovary, 4 leiomyosarcomas of the ovary and uterus, 8 ovarian sarcomas, one malignant teratoma, one sclerosing stromal tumor of the uterus and one uterine adenocarcinoma. The only case in which the *Brca1^Δ5-13^* and *p53^Δ2-10^* excision products were not detected was a peritoneal sarcoma that was not associated with the reproductive tract. Thus, conditional loss of both *Brca1* and *p53* in the ovary leads to a high frequency of ovarian and reproductive tract tumor formation.

**Table 2:** Summary of tumors associated with Cre-mediated inactivation of *Brca1* and/or *p53*.

Genotype	Total number of mice evaluated	Tumors with Cre-mediated conditional gene inactivation	Reproductive tract tumors with Cre-mediated conditional gene inactivation
***Brca1^Δ5-13^***	45	0	0
***p53^Δ2-10^***	47	2 (4%)	2 (4%)
***Brca1^Δ5-13^;p53^Δ2-10^***	41	23 (56%)	22 (54%)

### Histopathological Features of Tumors Arising in Mice with Conditionally Inactivated *Brca1* and/or *p53*

With the exception of one unclassifiable necrotic peritoneal mass, all of the tumors identified in *Brca1^LoxP/LoxP^* mice were classified as carcinomas. Among these was one vaginal squamous carcinoma, two subcutaneous adenocarcinomas located near the scapulae and five lung adenocarcinomas (not shown). PCR amplification of the excised *Brca1^Δ5-13^* product was not detected in any of these cases leading to the conclusion that these carcinomas were sporadic in nature. The relatively high frequency of sporadic tumor occurrence in this strain of mice remains unexplained.

In contrast to previous studies [22], [23], the ovaries of Ad5-CMV-Cre injected *Brca1^LoxP/LoxP^* mice were histologically unremarkable and did not exhibit noteworthy morphological or histological changes of the OSE indicative of pre-neoplastic or neoplastic growth. *Brca1^LoxP/LoxP^* mice that received bilateral intrabursal Ad5-CMV-Cre injections were observed for an average of 475 days (±102 days) prior to euthanasia suggesting that conditional inactivation of *Brca1* alone is insufficient to cause ovarian tumors even after long latency.

**Figure 3:**
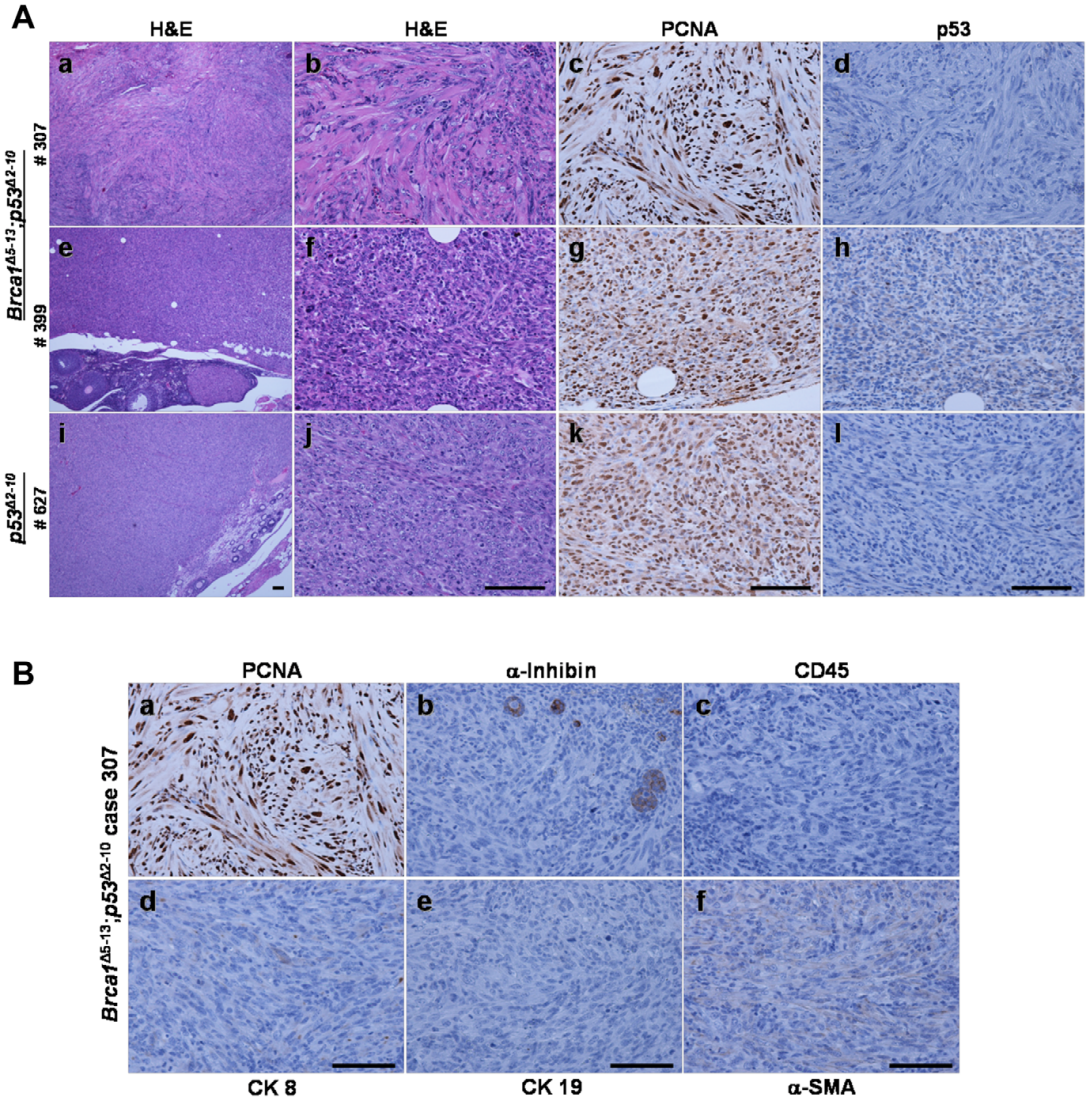
Histopathological characterization of ovarian tumors arising in *Brca1* ^Δ5-13^;*p53* ^Δ2-10^ and *p53^Δ2-10^* mice after intrabursal administration of Ad5-CMV-Cre. A) Low (panels a, e, and i) and high (panels b, f, and j) magnification of H&E stained sections of tumors isolated from two *Brca1* ^Δ5-13^;*p53* ^Δ2-10^ mice (cases number 307, panels a–d, and 399, panels e–h) and one *p53^Δ2-10^* mouse (case number 627, panels i-l). Immunohistochemical detection of proliferating cell nuclear antigen (PCNA) shows that all three tumors were highly proliferative (panels c, g, and k). Immunohistochemical staining for p53 (Panels d, h, and l) showing that none of the tumors express p53 protein, consistent with the conditional inactivation of the LoxP-flanked *p53* allele. B) Immunohistochemical staining of *Brca1* ^Δ5-13^;*p53* ^Δ2-10^ case number 307, a representative tumor, shows that the tumor is highly proliferative (PCNA staining, panel a), but does not express other common differentiation markers. Immunostaining for α-inhibin (panel b) shows isolated positively staining follicular cells and little or no staining of the tumor cells. Similarly, immunohistochemical detection of markers of lymphoid (CD45, panel c) epithelial (cytokeratins 8 and 19, panels d and e respectively) and stromal (α-smooth muscle actin, panel f) differentiation are not detected in tumor cells. Scale bars in each image represent 100 µm.

Histopathological evaluation of the single ovarian mass detected in a *p53^LoxP/LoxP^* mouse that received bilateral Ad-CMV-Cre injection showed that the tumor was an ovarian leiomyosarcoma (Figure 3A). The tumor was composed of spindle cells with marked cytologic atypia and numerous mitotic figures, including abnormal mitotic figures. The only other tumor detected in this group was a small uterine mass classified as a uterine leiomyosarcoma. In *Brca1^LoxP/LoxP^;p53^LoxP/LoxP^* mice that received bilateral Ad5-CMV-Cre injection, 23/41 (56%) mice developed confirmed *Brca1^Δ5-13^*;*p53^Δ2-10^*–associated tumors, most of which were ovarian. Among the ovarian tumors, the majority were categorized as soft tissue tumors including ovarian leiomyosarcomas (n = 12) and high grade sarcomas that could not be further classified (n = 4). These leiomyosarcomas exhibited spindle cells with marked atypia and the high grade sarcomas demonstrated pleiomorphic atypia without other specific morphologic features (Figure 3A). The ovarian tumors identified were highly proliferative as evidenced by nearly uniform PCNA staining (Figure 3A, panels c, g, and k) and, consistent with Cre-mediated inactivation of *p53^LoxP/LoxP^*, lacked expression of p53 (Figure 3A, panels d, h, and l). The ovarian tumors also lacked histopathological features or immunohistochemical staining of granulosa cell (α-inhibin), epithelial (CK8 and CK 19), lymphoid (CD3 and CD45R) or α-smooth muscle actin (α-SMA) differentiation (Figure 3B and data not shown). Thus, while conditional inactivation of both *Brca1* and *p53* resulted in a high frequency of ovarian tumor development, no ovarian carcinomas were detected.

### *Brca1* and *p53* Tumors Exhibit Genomic Instability

**Figure 4:**
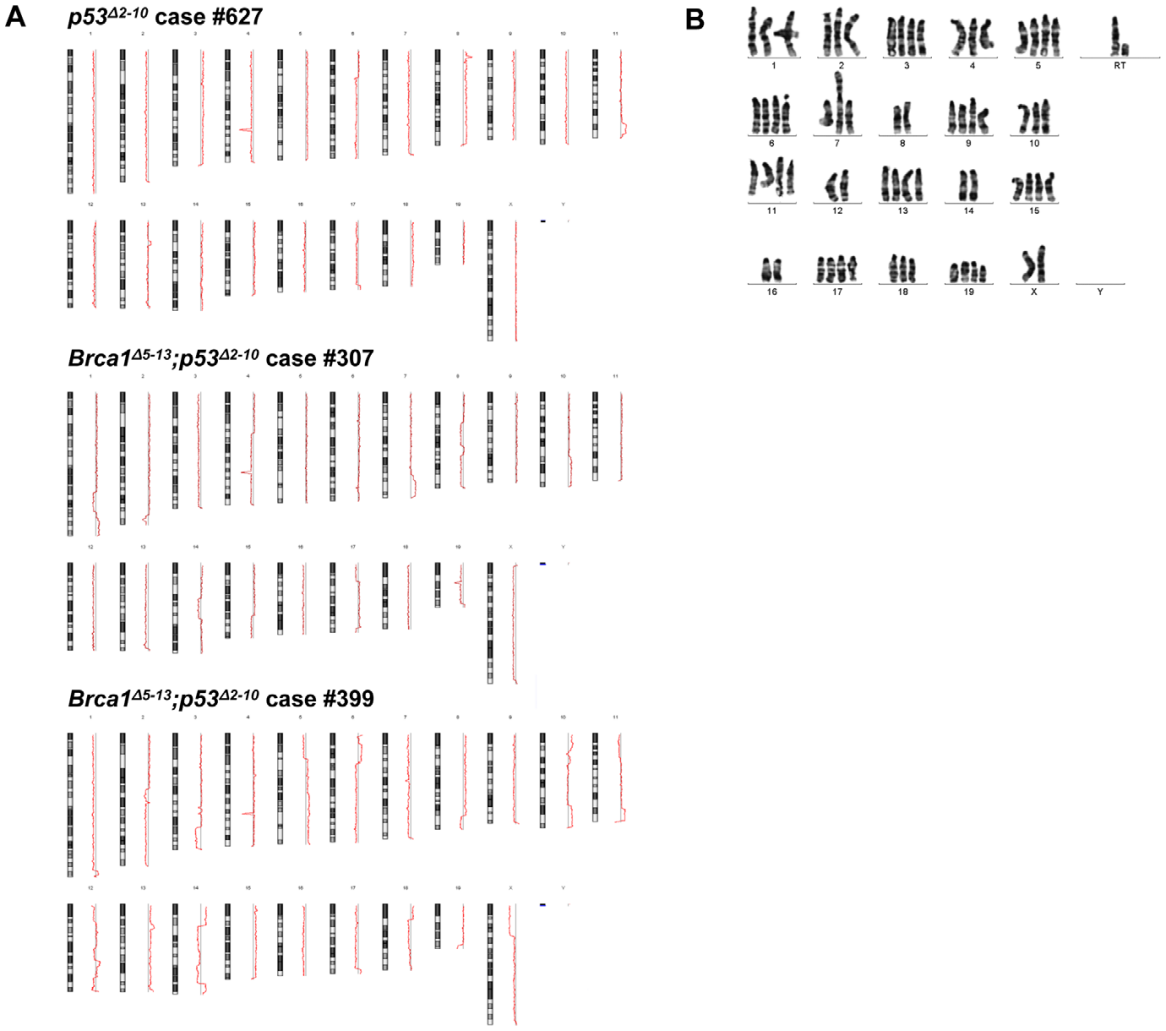
*Brca1* ^Δ5-13^;*p53* ^Δ2-10^ tumors exhibit a high degree of genomic instability. A) Array based comparative genomic hybridization was performed using genomic DNA isolated from *p53* ^Δ2-10^ (case #627) and *Brca1* ^Δ5-13^;*p53* ^Δ2-10^ (cases #307 and #399) ovarian tumors and shows that while *p53* ^Δ2-10^ tumors exhibit alterations consistent with aneuploidy, the *Brca1* ^Δ5-13^;*p53* ^Δ2-10^ ovarian tumors exhibit complex patterns of chromosomal copy number changes, with gains and losses apparent within the same chromosome. B) The high degree of genetic instability was verified by metaphase chromosome analysis of a cell line derived from *Brca1* ^Δ5-13^;*p53* ^Δ2-10^ tumor #399.

Genomic DNA isolated from the single *p53^Δ2-10^* ovarian leiomyosarcoma and two cases of *Brca1^Δ5-13^*;*p53^Δ2-10^* ovarian leiomyosarcomas was subjected to array based comparative genomic hybridization (aCGH) analysis. The *p53^Δ2-10^* ovarian leiomyosarcoma (case number 627) exhibited gains and/or losses of entire chromosomes analogous with aneuploidy and occasional isolated regions of DNA copy number alterations (Figure 4A). By comparison, the *Brca1^Δ5-13^*;*p53^Δ2-10^* ovarian leiomyosarcomas (case numbers 307 and 399) exhibited a much more complex array of genetic copy number alterations including entire chromosomal gains and losses as well as distinct regions of DNA gains and losses within the same chromosome (Figure 4A); results suggestive of a high degree of chromosomal damage. Karyotypic analysis of a tumor cell line established from *Brca1^Δ5-13^*;*p53^Δ2-10^* ovarian leiomyosarcoma case number 399 supported this observation. A total of 26 metaphase spreads were analyzed, 22 of which were karyotyped (Figure 4B). The number of chromosomes per individual metaphase ranged from 65 to 143, with no clear modal number. However, 18 metaphases were near-tetraploid, with chromosome counts ranging from 65–87. Seven metaphases had counts ranging from 97–143. The remaining metaphases showed a high level of chromosomal damage with many fragments, double minutes and a tri-radial rearrangement. Four of the metaphases had 1–3 chromosome or chromatid breaks, and 18 metaphases had one or more small markers or fragments. Thus, the cytogenetic analysis of this tumor cell line revealed considerable genomic instability. Due to the absence of *Brca1^Δ5-13^* tumors were unable to assess chromosomal stability in mice with conditional inactivation of *Brca1* alone; however, these results support the hypothesis that loss of *Brca1* function leads to a high degree of genetic instability thus contributing to ovarian tumorigenesis.

## Discussion

Although the lifetime risk of developing ovarian cancer is increased in women with germline mutations of *BRCA1*, previous studies in mice suggest that loss of *Brca1* alone was insufficient to lead to the development of EOC [22], [23], [25]. A number of independent studies [11], [12], [13], [14] have shown that in cases of EOC with *BRCA1* mutations, the frequency of mutations of *p53* is high, ranging from 70–90%. Moreover, in mouse models of mammary cancer, tumor latency is decreased and incidence of tumor formation increased in mice with both conditional inactivation of *Brca1* and a *p53* null allele [15]. This apparent cooperation of *Brca1* and *p53* in inherited breast and ovarian cancer in humans and in mammary tumor development in mice led us to create a mouse model of ovarian cancer by conditional inactivation of *Brca1* and *p53* in the ovarian epithelium.

Mice that are null or heterozygous for *p53* are highly prone to developing tumors, particularly lymphomas and sarcomas [33], [34]. For reasons that are not completely understood, the occurrence of epithelial tumors is relatively rare. More recent studies [27], [35] show that mice that have homozygous or heterozygous missense mutations of *p53* develop tumors with similar latency and spectrum to that of mice that are *p53* null or heterozygous, but that they also develop osteosarcomas and carcinomas with higher frequency. Missense mutations of *p53* have been shown to confer gain-of-function phenotypes compared to *p53* null mutations [27], [35]. Therefore, to develop a mouse model of *Brca1*- and *p53*-associated inherited EOC, we used two approaches for inactivation or mutation of *p53*. First, we predicted that using a strategy for tissue-restricted conditional inactivation of *p53^LoxP/LoxP^* in combination with *Brca1^LoxP/LoxP^* in the OSE would result in predisposition for the development of ovarian carcinomas. The second approach was based on the idea that missense mutations of *p53* confer gain-of-function phenotypes and possibly alter tissue specificity, favoring the development of carcinomas [27], [35]. Therefore, we also employed a mouse model expressing a missense mutation of *p53* (*p53^515A^*, analogous to the *p53* R175H hotspot mutation in human cancers [27]) in conjunction with *Brca1^LoxP/LoxP^*. Our results were consistent with previous findings in that ovary-specific conditional inactivation of either *Brca1* or *p53* alone rarely resulted in the development of invasive tumors [19], [22], [23], [25]. However, Cre-mediated conditional inactivation of both *Brca1* and *p53* resulted in the development of tumors in 56% (23/41) of the mice evaluated. Although mice heterozygous for the *p53^+/515A^* mutant allele develop tumors (i.e., lymphomas or sarcomas) with similar latency to *p53^+/−^* mice [27], we hypothesized that in mice that had also undergone Cre-mediated conditional inactivation of *Brca1*, ovarian carcinoma development might either out-pace or occur coincidentally with tumors at other sites. However, in spite of additional ovary-specific conditional inactivation of *Brca1*, tumors necessitating euthanasia developed at sites other than the ovary in *Brca1^LoxP/LoxP^;p53^+/515A^* and *Brca1^LoxP/LoxP^;p53^LoxP/515A^* mice. Histopathological evaluation revealed no ovarian or reproductive tract tumors in these mice. The strain of mice used in our study had been previously crossed with mice expressing Cre-recombinase under transcriptional control of the CMV gene promoter resulting in constitutive expression of the *p53^515A^* allele. It is therefore unknown whether mice would develop ovarian tumors if expression of *p53^+/515A^* or *p53^LoxP/515A^* were confined only to the OSE. Our results do suggest that ovarian tumor development in *Brca1^LoxP/LoxP^;p53^+/515A^* and *Brca1^LoxP/LoxP^;p53^LoxP/515A^* mice would require long latency and/or additional genetic alterations.

*BRCA1*-associated ovarian carcinomas in women typically exhibit serous histology [1]. Histopathological evaluation of the 22 *Brca1^Δ5-13^*;*p53^Δ2-10^* and two *p53^Δ2-10^* reproductive tract tumors unexpectedly showed a complex spectrum of tumors; ovarian leiomyosarcomas and sarcomas were most prevalent. In women, primary ovarian sarcomas are exceptionally rare [36], with very little known regarding the molecular alterations that contribute to their development. The reason for the lack of ovarian adenocarcinoma development in *Brca1^LoxP/LoxP^*;*p53^LoxP/LoxP^* mice given intrabursal injections of Ad5-CMV-Cre is unclear. Given the demonstration of highly selective ovarian surface epithelium infection after intrabursal administration of adenovirus, it seems unlikely to be due to technical issues associated with the injection procedure. The long latency between Ad5-CMV-Cre administration and the detection of tumors (∼400±100 days) and the high degree of genomic instability detected in tumors by aCGH and karyotyping analysis strongly suggests that additional events are required for ovarian tumorigenesis. This is consistent with previous findings [25] showing that *ex vivo* conditional inactivation of both *Brca1* and *p53* in ovarian explants was insufficient for transformation, whereas the addition of Myc cooperated to induce transformation in these cells. One possible explanation for the high incidence of ovarian leiomyosarcomas is that ovarian stromal cells may be coincidentally infected after intrabursal injection of Ad5-CMV-Cre and that the combined loss of both *Brca1* and *p53* renders this population of cells more susceptible to transformation than the ovarian surface epithelium. Consistent with this is the observation that *p53* null mice frequently develop sarcomas [33], [34]. Cre-mediated recombination of *p53^LoxP/LoxP^* alleles results in deletion of *p53* exons two through ten, thus rendering the cells and tissues undergoing recombination effectively *p53* null. Although this may potentially explain the high frequency of ovarian leiomyosarcomas observed, a previous study employing intrabursal adenovirus-Cre-recombinase mediated inactivation of LoxP-flanked sequences in *p53* ^LoxP/LoxP^;*Rb* ^LoxP/LoxP^ mice reported a high prevalence of ovarian carcinomas rather than leiomyosarcomas [19], suggesting the explanation may be more complicated than just relative tissue susceptibility in mice with *p53* mutations.

An alternative explanation for the lack of epithelial cancers in this model comes from several recent studies [37], [38], [39], [40] suggesting the precursor lesion of ovarian tumors arising in *BRCA1* mutation carriers may reside in the fallopian tube rather than the ovarian surface epithelium. These studies have nominated regions of distal fallopian tube mucosa exhibiting stabilized p53 protein, termed “p53 signatures”, and occult non-invasive intraepithelial serous carcinomas identified in prophylactic salpingo-oophorectomy specimens of *BRCA1* mutation carriers as candidate precursors of invasive ovarian and/or pelvic serous carcinomas. In fact, some suggest “p53 signatures” present in distal fallopian tube of non-*BRCA1* carriers as a plausible site of tumor origin for many sporadic ovarian and peritoneal cancers [41]. This may suggest that in attempting to model inherited EOC, the relevant target tissue for recombination and excision of *Brca1* and *p53* may not be the OSE, but rather the epithelium of the distal fallopian tube. Based on the anatomy of murine distal fallopian tube and ovary, intrabursal injection of Ad5-CMV-Cre would be predicted to result in viral contact with at least a portion of the tubal fimbria. This prediction is supported by our observation of isolated β-galactosidase positive cells present in the fallopian tube after intrabursal injection of Ad5-CMV-LacZ. However, this strategy appears to result in less frequent recombination in the fallopian tube than the OSE. Interestingly, in a very recent study that used a genetic approach for reproductive tract restricted Cre-recombinase expression by crossing Amhr2-Cre transgenic mice with *Brca1^LoxP/LoxP^* and/or *p53^LoxP/LoxP^* mice showed that while Cre-mediated recombination of floxed *Brca1* and *p53* alleles could be demonstrated in the ovaries, fallopian tubes and uteri of compound transgenic mice, the tumors that developed in these mice were all uterine leiomyosarcomas rather than ovarian tumors of any type [42]. Further studies will likely elucidate whether secretory epithelial cells of the tubal fimbria are the primary cell of origin of *BRCA1*-associated and/or sporadic serous ovarian carcinomas. If this is the case, an alternate strategy for development of mouse models of inherited *Brca1*-associated, and possibly sporadic serous EOC, would be to develop a transgenic strain of mice that express the *Cre-recombinase* gene under transcriptional control of a gene expressed selectively in the fallopian tube epithelium, such as the *oviduct-specific glycoprotein 1* gene [43], [44].

Unlike other tumor suppressor genes, loss or mutation of *BRCA1* confers a selective risk for cancer development in organs such as breast, ovary and prostate. The mechanisms of enhanced tissue-specific risk are unclear, but suggest that production of or responsiveness to hormones may play an important role. Recent work suggests that *BRCA1* inactivation contributes to ovarian tumor development by cell non-autonomous mechanisms [22]. In this study, investigators used a Cre/LoxP strategy to specifically inactivate *Brca1* in the granulosa cells of the mouse ovary by crossing *Brca1^LoxP/LoxP^* mice to transgenic mice expressing Cre-recombinase under transcriptional control of the follicle stimulating hormone receptor (*Fshr*) gene promoter. Otherwise genetically normal female mice with *Brca1* inactivated only in granulosa cells developed benign epithelial ovarian neoplasms [22]. Analysis of microdissected epithelial lesions confirmed that no excision of the LoxP-flanked *Brca1* allele occurred in these cells, suggesting that restricted conditional inactivation of *Brca1* in granulosa cells is sufficient to induce neoplastic alterations in the epithelial compartment in a cell non-autonomous manner. Recent evidence suggests an additional role for *BRCA1* in the regulation of aromatase P450 (CYP19), the enzyme that catalyzes conversion of androgen to estrogen [45]. Results from this study suggest that *BRCA1* mutation and loss of heterozygosity may predispose cancer formation in steroidogenic tissues by virtue of loss of DNA damage repair mechanisms in the epithelial cell compartments coupled with growth stimulatory paracrine effects on these cells mediated by enhanced estrogen production in local tissues (i.e., granulosa cells and/or preadipocytes). Other studies suggest that selective inactivation of *BRCA1* or *p53* in the stroma surrounding tumors plays a direct role in tumor progression in steroidogenic organs [46], [47], [48]. Interestingly, recent work showed that cells exhibiting a senescence associated secretory phenotype (SASP) contribute to epithelial to mesenchymal transitions and invasiveness in a cell non-autonomous manner, with inactivation of *p53* being a major contributor to these pro-malignant paracrine activities [49]. It is well-established that genetic and/or epigenetic alterations within the underlying tumor stroma have a direct effect on tumor progression (reviewed in [50]). Hence, it is clear that stromal-epithelial interactions are critical to both normal ovarian tissue function and to epithelial tumor initiation and progression in this hormone responsive organ.

Taken together, previously published studies and our own data suggest that loss of function of *BRCA1* and *p53* cooperate in their contribution to ovarian tumorigenesis, but that additional molecular or epigenetic alterations within tumor cells and/or stroma as well as cell non-autonomous factors contribute to the development of EOC. Further analyses are required to elucidate the mechanisms of BRCA1-mediated transformation of the ovarian epithelium.

## Materials and Methods

### Mutant Mouse Strains

All procedures in this study involving mice were approved by the Fox Chase Cancer Center Institutional Animal Care and Use Committee (IACUC). Mice harboring LoxP flanked sequences in *Brca1* (FVB;129-*Brca1^tm2Brn^*) [16] and *p53* (FVB;129-*Trp53^tm1Brn^*) [26] were obtained from the Mouse Models of Human Cancer Consortium (MMHCC) Mouse Repository (http://mouse.ncifcrf.gov/, National Cancer Institute, Frederick, MD) and maintained as homozygous colonies expressing the conditional alleles (*Brca1^LoxP/LoxP^* and *p53^LoxP/LoxP^*). Mice harboring an arginine to histidine substitution at amino acid 172 of *p53* (*p53^515A/515A^*) [27], a mutation corresponding to the *p53* hot spot mutation at Arg 175 in humans [28], [29], [30], were generously provided by Dr. Guillermina Lozano, University of Texas MD Anderson Cancer Center, Houston, TX). Mice harboring conditional (LoxP flanked) alleles of *Brca1* or *p53* or mutant *p53* (*Brca1^LoxP/LoxP^*, *p53^LoxP/LoxP^* and/or *p53^515A/515A^*) were intercrossed to generate colonies with the following compound genotypes: *Brca1^LoxP/LoxP^;p53^LoxP/LoxP^*, *Brca1^LoxP/LoxP^;p53^+/515A^* and *Brca1^LoxP/LoxP^;p53^LoxP/515A^*.

### Preparation of Genomic DNA and Genotyping Analysis

Genomic DNA was isolated from tail biopsies by overnight digestion in sodium chloride (NaCl)/Tris EDTA/proteinase K solution at 55°C and ethanol precipitation as previously described [17]. Mice harboring the *Brca1^LoxP/LoxP^* allele were genotyped as described [16], [23] using primers flanking introns 4 (*Brca1*-int4-Fwd-5′-TATCACCACTGAATCTCTACCG -3′ and *Brca1*-int4-Rev-5′-GACCTCAAACTCTGAGATCCAC-3′) and 13 (*Brca1*-int13-Fwd-5′-TATTCTTACTTCGTGGCACATC-3′ and *Brca1*-int13-Rev-5′-TCCATAGCATCTCCTTCTAAAC-3′). The expected sizes of wild type and LoxP flanked *Brca1* sequences were 390 nt and 545 nt for intron 4 and 494 nt and 620 nt for intron 13 respectively. Mice harboring the *p53^LoxP/LoxP^* allele were genotyped as previously described [26] using primers flanking introns 1 (*p53*-int1-Fwd-5′-CACAAAAACAGGTTAAACCAG-3′ and *p53*-int1-Rev-5′-AGCACATAGGAGGCAGAGAC-3′) and 10 (*p53*-int10-Fwd-5′-AAGGGGTATGAGGGACAAGG-3′ and *p53*-int10-Rev-5′-GAAGACAGAAAAGGGGAGGG-3′). The expected sizes of wild type and LoxP flanked *p53* sequences were 288 nt and 370 nt for intron 1 and 431 nt and 584 nt for intron 10 respectively. Mice harboring mutant *p53^515A^* allele(s) were genotyped as previously described [51] using primers PL5-5′-ACCTGTAGCTCCAGCACTGG-3′ , PL6-5′-ACAAGCCGAGTAACGATCAGG-3′ and NeoR-5′-CCATTTGTCACGTCCTGCACG-3′ .

Genomic DNA was isolated from bulk tumor tissue using the same method as described above. Alternatively, microscopic tumors were manually microdissected. Briefly, adjacent H&E stained sections were aligned with unstained paraffin embedded tissue sections to identify the area containing the tumor lesion which was then scraped with needles or scalpel blades as described [52], [53]. The microdissected tissue was then deparaffinized in xylenes and genomic DNA isolated by standard methods for phenol/chloroform/ethanol extraction [52], [53]. Purified genomic DNA was used for PCR amplification and detection of LoxP-flanked and excised *Brca1^LoxP/LoxP^*, *p53^LoxP/LoxP^* alleles. Cre recombinase-mediated excision of LoxP flanked sequences in *Brca1^LoxP/LoxP^* mice results in deletion of exons 5–13 (*Brca1^Δ5-13^*) and was detected by PCR amplification of genomic DNA with the *Brca1*-int4-Fwd and *Brca1*-int13-Rev primers. Detection of the 594 nt *Brca1^Δ5-13^* PCR product confirmed excision. Similarly, Cre-mediated excision of LoxP flanked sequences in *p53^LoxP/LoxP^* mice resulting in deletion of exons 2–10 (*p53^Δ2-10^*) was detected by amplification of a 612 nt PCR product from genomic DNA amplified with the *p53*-int1-Fwd and *p53*-int10-Rev primers.

### Adenovirus Administration

Replication defective recombinant adenoviruses expressing *LacZ* (Ad5-CMV-ntLacZ) or Cre-recombinase (Ad5-CMV-Cre) were purchased from the Gene Transfer Vector Core, University of Iowa, Iowa City, IA. To demonstrate our capability to successfully perform intrabursal injections, we performed pilot experiments in which 8 week-old female C57Bl/6 mice received intrabursal injections of Ad5-CMV-ntLacZ as previously described [18], [19], [21], [23], [54]. To synchronize ovulation, female mice were injected intraperitoneally (i.p.) with 5 U of pregnant mare serum gonadotropin, followed 48 hours later by i.p. injection with 5 U of human chorionic gonadotropin (hormones purchased from Sigma, St. Louis, MO). In preparation for surgery, mice were anesthetized by intraperitoneal injection of 95 µL per 10 gram body weight of 10 mg/mL Ketamine hydrochloride solution and 1 mg/mL Xylazine hydrochloride 36 hours following the last hormone injection. The ovaries were accessed and exposed through a dorsal incision and each ovary received a single intrabursal injection of approximately 10 µL (5×10^7^ plaque forming units, p.f.u.) of Ad5-CMV-ntLacZ virus by inserting a 32G needle coupled with a Hamilton syringe through the oviduct near the infundibulum into the ovarian bursa.

For Cre-mediated excision of LoxP-flanked *Brca1^LoxP/LoxP^* and/or *p53^LoxP/LoxP^* alleles, mice received intrabursal injections of Ad5-CMV-Cre-recombinase (5×10^7^ p.f.u.) as described above for Ad5-CMV-ntLacZ. Mice were subjected to bilateral or unilateral intrabursal injection of Ad5-CMV-Cre or unilateral injection of PBS (controls) and monitored daily for wellness for the first ten days post-surgery. Thereafter, mice were monitored at least twice a week for wellness and the development of palpable tumors. Once tumors were detected, mice were monitored daily until euthanasia. Mice were euthanized by CO_2_ asphyxiation if estimated tumor palpable tumor volume reached 10% of the total body weight or if mice exhibited any signs of loss-of-wellness, as per IACUC guidelines.

### Detection of Ad5-CMV-ntLacZ Infection and Ad5-CMV-Cre-Mediated Excision

Mice infected with Ad5-CMV-ntLacZ were euthanized 7 days after intrabursal injection. Ovaries were removed, embedded in OCT medium (Tissue-Tek), snap frozen in liquid N_2_ and 10 µm sections cut with a cryostat. Sections were fixed for 10 minutes in 0.5% glutaraldehyde, followed by two rinses with PBS+0.1% Tween-20, then stained with X-gal solution (1 mg/mL X-gal (Promega, Madison, WI), 5 mM K_3_Fe(CN)_6_, 5 mM K_4_Fe(CN)_6_, 1 mM MgCl_2_ in phosphate-buffered saline (PBS)) at 37°C overnight in a humidified chamber. Sections were rinsed twice in PBS+0.1% Tween-20, counterstained with Nuclear Fast Red, dehydrated with alcohols, cleared in xylenes, and coverslipped using Permount (Fisher).

Tissue restricted Ad5-CMV-Cre infection and Cre-mediated excision of LoxP flanked alleles was confirmed by isolation of genomic DNA from ovary, uterus and liver tissue excised from mice seven days after intrabursal injection of Ad5-CMV-Cre. Tissues were digested in NaCl/Tris EDTA/proteinase K solution at 55°C and genomic DNA isolated as described [17]. A tissue lysate enriched for digested OSE was prepared by limiting the initial digestion time of ovary specimens at 55°C to 30 min. After the first 30 min., the samples were centrifuged briefly at low speed (1000 rpm) to pellet the undigested ovary. The supernatant was collected and genomic DNA was immediately isolated. To continue digestion of the remainder of the ovary, a fresh aliquot of NaCl/Tris-EDTA/proteinase K digestion buffer was added and the sample digested overnight along with other tissue specimens. Genomic DNA was isolated the following day and subjected to PCR amplification with *Brca1* intron 4 and intron 13 primers for detection of LoxP flanked and excised sequences as described above.

### Preparation and Analysis of Tissues, Histology and Immunohistochemistry

All mice were euthanized by CO_2_ asphyxiation, necropsied and examined for gross abnormalities. Pathologically altered organs, entire reproductive tracts and representative specimens of multiple organs and tissues, including the brain, lung, liver, kidney, spleen, pancreas and intestine were removed at necropsy, fixed in 10% (v/v) neutral buffered formalin (NBF) overnight, transferred to 70% ethanol and paraffin-embedded. In mice with evident tumor, specimens of the tumor tissue were also excised, snap frozen in liquid N_2_ and stored at −80°C. For histological analysis, 5 µm formalin fixed paraffin embedded tissue sections were cut for either H&E staining or immunohistochemistry (IHC). Histopathological analysis was performed by a Board Certified Surgical Pathologist with expertise in human and murine gynecological malignancies (LHE).

Sections of tumor tissue for immunohistochemical (IHC) staining were cut on SuperFrost Plus charged slides (Fisher). Antigens (dilution and source of primary antibodies indicated in parenthesis) detected by IHC included: Proliferating nuclear antigen (PCNA, 1∶1000; Biogenex), p53 (1∶400; Vector Labs), cytokeratin 8 and 19 (CK8, 1∶100 and CK19, 1∶50; Developmental Studies Hybridoma Bank, The University of Iowa), CD3 (1∶100; DAKO), CD45R/B220 (1∶200; Pharmingen), α-smooth muscle actin (α-SMA, 1∶100; Sigma) and α-inhibin (1∶50; Serotec). Unstained sections were deparaffinized, subjected to antigen retrieval and stained as described [17].

### Establishment of Ovarian Cancer Cell Lines and Cell Culture

To establish a cell line from an ovarian tumor, approximately 1 cm^3^ fragments of tumor were excised under aseptic conditions, transferred to a 10 cm^2^ cell culture dish and finely minced using sterile scalpel blades. Minced tumor tissue was further disaggregated by passing several times through a syringe coupled with a 21½ G needle. The suspension of minced tumor tissue was plated directly in DMEM supplemented with 4% FBS, 1X insulin/transferring/selenium (ITS), penicillin/streptomycin (100 units/mL and 2 mM l-glutamine, and incubated at 37°C in 5% CO_2_. The culture medium was changed twice weekly until the cells reach confluence at which time they were trypsinized and passaged at 4–5 day intervals.

### Array CGH and Karyotype Analysis

For aCGH, high quality genomic DNA (0.5 to 3 µg) was isolated from frozen tumor specimens using standard methods and digested with restriction endonucleases *Alu*I and *Rsa*I according to the Agilent Oligonucleotide Array-based CGH for Genomic DNA Analysis Version 4.0 protocol. The digested genomic DNA was labeled using Agilent Genomic DNA Labeling Kit PLUS. Test and reference DNA samples were labeled with either cyanine 5- or cyanine 3-dUTP, according to the manufacturer's instructions. Cyanine 5- and cyanine 3-labeled DNA products were then purified using Microcon YM-30 (Millipore) filtration devices. The DNA yield and level of dye incorporation were measured using the ND-1000 Spectrophotometer. Appropriate cyanine 5- and cyanine 3-labeled DNA sample pairs were combined and then mixed with mouse Cot-1 DNA, Agilent 10X Blocking Agent, and Agilent 2X Hybridization Buffer. The labeled target solution was hybridized to Agilent 244K Mouse Genome CGH microarray (G4415A) using SureHyb chambers. After hybridization the microarrays were washed and dried according to the procedures described in the manufacturer's protocol. Microarray slides were scanned immediately using an Agilent microarray scanner. Data for individual features on the microarray were extracted from the scan image using Agilent Feature Extraction (FE) Software. Output files from FE were imported into Agilent CGH data analysis software, CGH Analytics for DNA copy number analysis.

A tumor cell line established from a *Brca1^Δ5-13^*;*p53^Δ2-10^* ovarian leiomyosarcoma was subjected to karyotype analysis. Preparation of metaphase spreads and trypsin-Giemsa (G) banding were performed according to standard procedures. Chromosome identification and karyotypes designations were in accordance with the University of Washington guidelines (http://www.pathology.washington.edu/research/cytopages/idiograms/mouse/).

### Statistical Analysis

The Kaplan-Meier method was used to construct survival plots for each data set. Comparisons of each of the data sets were made using the Log-Rank test.
